# *AtC3H3*, an *Arabidopsis* Non-TZF Gene, Enhances Salt Tolerance by Increasing the Expression of Both ABA-Dependent and -Independent Stress-Responsive Genes

**DOI:** 10.3390/ijms252010943

**Published:** 2024-10-11

**Authors:** Hye-Yeon Seok, Sun-Young Lee, Linh Vu Nguyen, Md Bayzid, Yunseong Jang, Yong-Hwan Moon

**Affiliations:** 1 Institute of Systems Biology, Pusan National University, Busan 46241, Republic of Korea; seokhyeon@pusan.ac.kr (H.-Y.S.); symoonlee@pusan.ac.kr (S.-Y.L.); 2 Department of Animal and Aquatic Sciences, Faculty of Agriculture, Chiang Mai University, Chiang Mai 50200, Thailand; linhvu.n@cmu.ac.th; 3 Department of Integrated Biological Science, Pusan National University, Busan 46241, Republic of Korea; md.bayzid18fvm@gmail.com (M.B.); seong9466@gmail.com (Y.J.); 4 Department of Molecular Biology, Pusan National University, Busan 46241, Republic of Korea

**Keywords:** ABA-dependent pathway, ABA-independent pathway, *AtC3H3*, CCCH zinc-finger, non-TZF, salt stress

## Abstract

Salinity causes widespread crop loss and prompts plants to adapt through changes in gene expression. In this study, we aimed to investigate the function of the non-tandem CCCH zinc-finger (non-TZF) protein gene *AtC3H3* in response to salt stress in *Arabidopsis*. *AtC3H3*, a gene from the non-TZF gene family known for its RNA-binding and RNase activities, was up-regulated under osmotic stress, such as high salt and drought. When overexpressed in *Arabidopsis*, *AtC3H3* improved tolerance to salt stress, but not drought stress. The expression of well-known abscisic acid (ABA)-dependent salt stress-responsive genes, namely *Responsive to Desiccation 29B* (*RD29B*), *RD22*, and *Responsive to ABA 18* (*RAB18*), and representative ABA-independent salt stress-responsive genes, namely *Dehydration-Responsive Element Binding protein 2A* (*DREB2A*) and *DREB2B*, was significantly higher in *AtC3H3*-overexpressing transgenic plants (*AtC3H3* OXs) than in wild-type plants (WT) under NaCl treatment, indicating its significance in both ABA-dependent and -independent signal transduction pathways. mRNA-sequencing (mRNA-Seq) analysis using NaCl-treated WT and *AtC3H3* OXs revealed no potential target mRNAs for the RNase function of AtC3H3, suggesting that the potential targets of AtC3H3 might be noncoding RNAs and not mRNAs. Through this study, we conclusively demonstrated that *AtC3H3* plays a crucial role in salt stress tolerance by influencing the expression of salt stress-responsive genes. These findings offer new insights into plant stress response mechanisms and suggest potential strategies for improving crop resilience to salinity stress.

## 1. Introduction

Salinity is increasingly contributing to global crop losses. Gene expression systems play a crucial role in altering plant metabolism, as well as regulating cell growth, division, and differentiation, all of which are necessary for stress adaptation [1]. At least two pathways participate in transmitting osmotic stress caused by high salinity or drought stress. One pathway is abscisic acid (ABA)-dependent, whereas the other is ABA-independent [2,3]. The ABA-dependent pathway relies on elevated cellular ABA levels caused by osmotic stress, activating osmotic stress-responsive genes such as *Responsive to Desiccation 29B* (*RD29B*), *Responsive to ABA 18* (*RAB18*), *RD20*, and *RD22* [2,3]. In the ABA-independent pathway, osmotic stress-responsive gene activation does not require ABA up-regulation. Instead, osmotic stress-responsive genes are activated through C-repeat/dehydration-responsive elements (CRT/DRE) in their promoters, along with associated CRT/DRE-binding factors (CBF/DREBs), such as Dehydration-Responsive Element Binding protein 2A (DREB2A) and DREB2B. During osmotic stress, both signaling pathways maintain cellular homeostasis by up-regulating specific genes [2,3].

Zinc-finger proteins with a CCCH zinc-finger motif comprising three cysteine (Cys) residues and one histidine (His) residue are known as CCCH zinc-finger proteins. These proteins have been detected in most eukaryotic organisms and play crucial roles in plant biology [4]. In *Arabidopsis* (*Arabidopsis thaliana*), 68 CCCH zinc-finger protein genes are known, whereas 67 were found in rice (*Oryza sativa*). *Arabidopsis* CCCH zinc-finger proteins are categorized into 11 subfamilies depending on the number of zinc-finger motifs and the distance between the Cys and His residues [4]. Additionally, CCCH zinc-finger proteins are categorized as tandem and non-tandem CCCH zinc-finger (TZF and non-TZF, respectively) proteins. TZF proteins have two tandem CCCH zinc-finger motifs, while non-TZF proteins contain a different number of CCCH zinc-finger motifs. The *Arabidopsis* genome contains 26 presumptive TZF and 42 non-TZF proteins [5].

The CCCH zinc-finger proteins play essential roles in various biological processes in plants, such as responding to hormones and stresses, providing immunity against pathogens, maintaining homeostasis, and regulating plant growth [6]. Previous research has shown that specific CCCH zinc-finger protein-encoding genes regulate abiotic stress responses. For example, *AtTZF10*/*Salt-inducible Zinc Finger 2* (*AtSZF2*) and *AtTZF11*/*AtSZF1* are involved in the negative salt stress response, while *AtTZF4*/*SOMNUS* (*SOM*), *AtTZF5*, and *AtTZF6*/*PEI1* are involved in the positive ABA response [7,8]. In *Arabidopsis*, *AtTZF2*/*Oxidation-related Zinc Finger 1* (*AtOZF1*) or *AtTZF3*/*AtOZF2* overexpression enhances drought tolerance and hypersensitive responses to ABA [9]. Additionally, *AtC3H17*, a non-TZF gene in *Arabidopsis*, is involved in osmotic stress responses, with increased expression in response to high salt and drought stress. *AtC3H17* overexpression confers resistance to high salt and oxidative stress, whereas mutants show higher sensitivity than wild-type plants (WT). Furthermore, *AtC3H17* is involved in high salt stress resistance in *Arabidopsis* through an ABA-dependent pathway [10]. In rice, *OsTZF1* is up-regulated by high salinity, drought, and oxidative stress [11]. *GhZFP1* overexpression improves drought tolerance in cotton (*Gossypium hirsutum*) [12]. *IbC3H18*, a non-TZF gene in sweet potato (*Ipomoea batatas*), and *TaZnFP*, identified in wheat (*Triticum aestivum*), are up-regulated by salt and drought stresses. *IbC3H18* or *TaZnFP* overexpression improves salt and drought stress tolerance [13,14]. Plants overexpressing *DgC3H1*, a non-TZF gene in chrysanthemum (*Chrysanthemum morifolium*), showed greater tolerance to low-temperature stress. In contrast, low-temperature stress tolerance was reduced in antisense *DgC3H1*-expressing transgenic plants [15].

Zinc-finger proteins are essential for various cellular processes, including RNA binding, transcriptional regulation, protein–protein interactions, and apoptosis regulation [16]. Some CCCH zinc-finger proteins are located in the cytoplasm, bind to RNA, regulate RNA stability, and post-transcriptionally control downstream genes. Certain CCCH zinc-finger proteins include nuclear localization and/or nuclear export signals, working as transcription factors in the nucleus by activating or repressing transcription [6]. Therefore, CCCH zinc-finger proteins control the transcriptional and post-transcriptional expression of development- or stress response-associated genes. CCCH zinc-finger proteins mediate post-transcriptional regulation through their function as RNA-binding proteins. For instance, AtTZF1 uses its TZF motif to bind to RNA in a zinc-dependent manner [17]. In *Arabidopsis*, AtC3H11/Cleavage and Polyadenylation Specificity Factor 30 (AtCPSF30) is essential for cleaving the 3′ end of pre-mRNA and generating the poly-A tail. Notably, the position of the 3′ end can vary owing to AtC3H11/AtCPSF30 [18]. It has been demonstrated that plant CCCH zinc-finger proteins control transcription. AtC3H14 and AtC3H15/Callose Defective Microspore 1 (AtCDM1) exhibit transactivation activity as well as DNA binding in yeast [19]. In *Arabidopsis*, AtC3H17 contains an EELR-like motif at its N-terminus, which activates downstream gene transcription [5]. IbC3H18 possesses an RNA-binding motif and exhibits transactivation activity. The C-terminus contains an RNA-binding motif that is crucial for transcriptional activation [14]. The LlC3H18 transcription factor with an RNA-binding function in lily (*Lilium longiflorum*) activates transcription and regulates responses to high-temperature stress by binding to the *LlWRKY33* promoter [20].

In this study, we investigated the role of *AtC3H3*, a non-TZF gene, in the response of *Arabidopsis* to salt stress. AtC3H3, known for its RNA-binding and RNase activities [21], is up-regulated under osmotic stress, particularly high salt and drought. Our study revealed that *AtC3H3*-overexpressing *Arabidopsis* exhibited tolerance to salt stress. Furthermore, *AtC3H3* was responsible for the response of *Arabidopsis* to salt stress by influencing both ABA-dependent and -independent pathways.

## 2. Results

### 2.1. AtC3H3 Possesses Five CCCH Zinc-Finger Motifs

**Figure 1:**
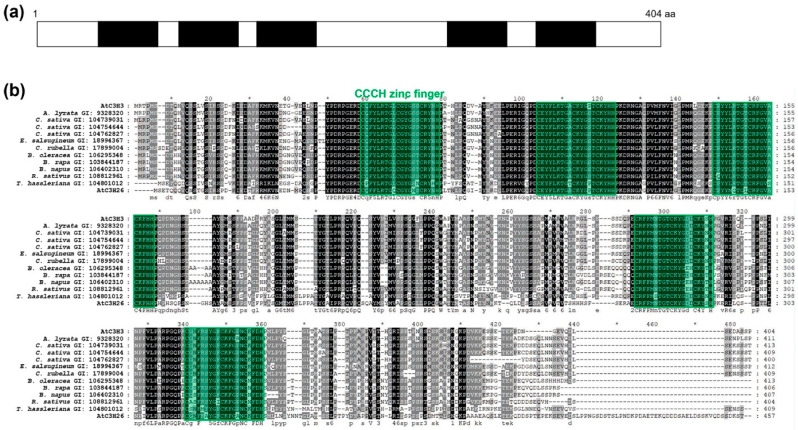
Domain structure of AtC3H3 and multiple sequence alignments among AtC3H3, its paralog, and its orthologs. (**a**) Black boxes indicate CCCH zinc-finger motifs in AtC3H3. (**b**) Multiple sequence alignment with protein sequences of AtC3H3, its paralog, and orthologs. Green boxes indicate CCCH zinc-finger motifs conserved among AtC3H3, its paralog, and its orthologs. Conservation rates of amino acids are represented by shading: black shade for 100%, dark gray shade for 80%, and light gray shade for 60%. GI number of each protein sequence is as follows: AtC3H3, 839351; *A. lyrata*, 9328320; *C. sativa*, 104739031; *C. sativa*, 104754644; *C. sativa*, 104762827; *E. salsugineum*, 18994367; *C. rubella*, 17899004; *B. oleracea*, 106295348; *B. rapa*, 103844187; *B. napus*, 106402310; *R. sativus*, 108812961; *T. hassleriana*, 104801012; AtC3H26, 817855.

To better understand osmotic stress responses mediated by non-TZF genes, we analyzed the expression of non-TZF genes under osmotic stress and selected *AtC3H3* as a candidate for further study. AtC3H3 has five CCCH zinc-finger motifs, denoted as C–X_8_–C–X_5_–C–X_3_–H, and is included in subfamily I of CCCH zinc-finger proteins in *Arabidopsis* (Figure 1). We identified one AtC3H3 paralog, AtC3H26, and several AtC3H3 orthologs in several plant species, including *Arabidopsis lyrata*, *Camelina sativa*, *Eutrema salsugineum*, *Capsella rubella*, *Brassica oleracea*, *Brassica rapa*, *Brassica napus*, *Raphanus sativus*, and *Tarenaya hassleriana* by BLASTP analysis. Multiple sequence alignment of AtC3H3 and its paralog and orthologs revealed highly conserved protein sequences, particularly in the CCCH zinc-finger motifs (Figure 1b).

### 2.2. AtC3H3 Expression during Development and in Organs in Arabidopsis

**Figure 2:**
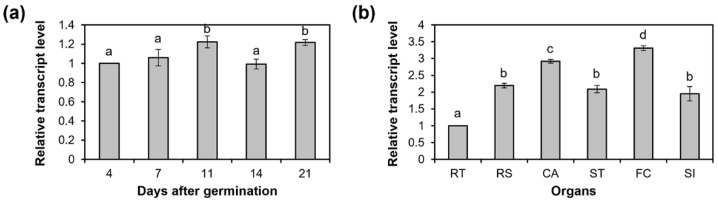
*AtC3H3* expression patterns during seedling development and in mature plant organs using quantitative RT-PCR (qRT-PCR). (**a**) Relative transcript levels of *AtC3H3* at different developmental stages in wild-type plant (WT) seedlings grown under short-day (SD) conditions. (**b**) Relative transcript levels of *AtC3H3* in organs of 49 days after germination (DAG) WT grown under long-day (LD) conditions. RT, roots; RS, rosette leaves; ST, stems; CA, cauline leaves; FC, floral clusters; SI, siliques. In (**a**,**b**), *GAPc* was used to normalize the relative transcript levels. Data represent the average with standard deviations indicated by error bars (*n* = 3). Statistical differences (*p* < 0.05) are denoted by different letters.

To obtain insights into the functions of *AtC3H3*, the temporal and spatial patterns of *AtC3H3* expression were investigated at various seedling developmental stages and in mature plant organs using quantitative RT-PCR (qRT-PCR). *AtC3H3* showed constitutive transcript levels during *Arabidopsis* seedling development (Figure 2a). In mature *Arabidopsis* plants, *AtC3H3* was significantly transcribed in floral clusters and cauline leaves compared to the other organs investigated, including roots, siliques, stems, and rosette leaves (Figure 2b). In the semi-qRT-PCR analysis, we observed similar expression patterns to those obtained through qRT-PCR (Figure S1).

**Figure 3:**
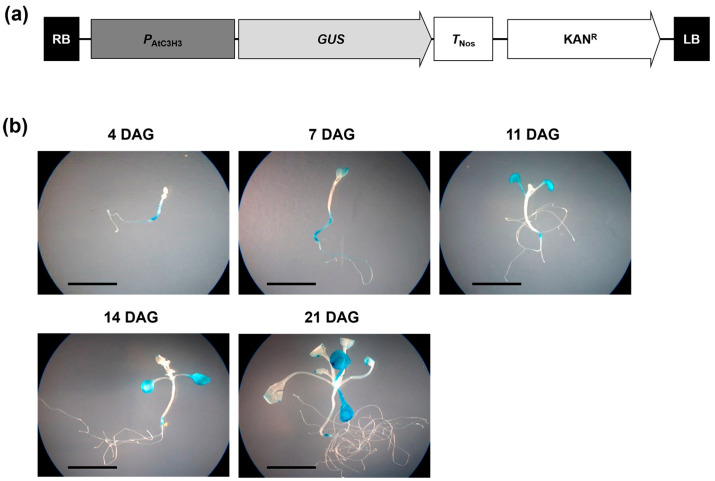
Promoter activity of *AtC3H3*. (**a**) Schematic map of *β-glucuronidase* (*GUS*)-fused *AtC3H3* promoter construct. (**b**) Histochemical GUS assay conducted using transgenic plants harboring *GUS*-fused *AtC3H3* promoter construct grown under SD conditions for indicated times. Scale bars indicate 1 cm.

To monitor the *AtC3H3* expression patterns, we generated transgenic plants harboring a *β-glucuronidase* (*GUS*)-fused *AtC3H3* promoter construct and examined them using a histochemical GUS assay. The 2 kb upstream region from the transcription initiation site with 180 bp of *AtC3H3* 5′ UTR was linked to *GUS* (Figure 3a). *GUS* expression was observed in the cotyledons and root junctions of 4- to 21-day-old seedlings, and it was constitutively expressed throughout *Arabidopsis* seedling development (Figure 3b), indicating that the *AtC3H3* promoter was activated constitutively during *Arabidopsis* seedling development. This result was in line with the *AtC3H3* expression pattern observed using qRT-PCR (Figure 2a).

### 2.3. Subcellular Localization of AtC3H3 Protein

**Figure 4:**
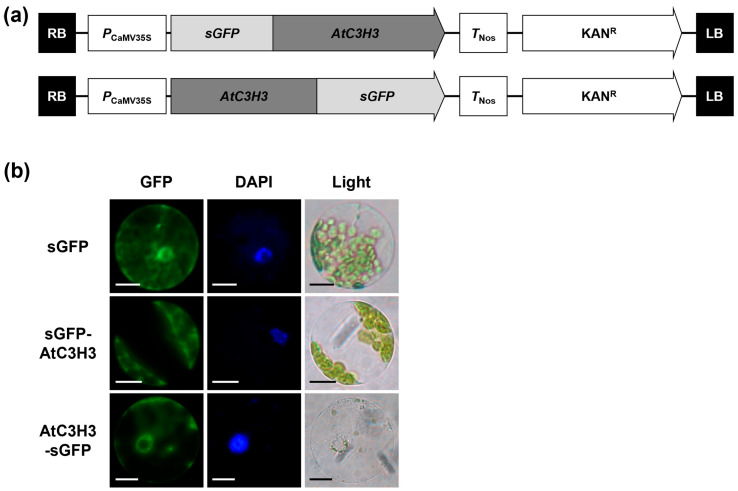
Subcellular AtC3H3 localization. (**a**) Schematic maps of N-terminal or C-terminal sGFP-fused AtC3H3 constructs. (**b**) Subcellular AtC3H3 localization investigated by transiently expressing sGFP–AtC3H3 and AtC3H3–sGFP constructs in *Arabidopsis* protoplasts. Left, GFP signal; middle, 4′,6-diamidino-2-phenylindole (DAPI) staining; right, light microscopic image. Scale bars indicate 10 μm.

To reveal the molecular actions of AtC3H3, we examined the subcellular localization of AtC3H3 using the synthetic GFP (sGFP)-fused AtC3H3 constructs expressed in *Arabidopsis* protoplasts (Figure 4a). The GFP signals of both sGFP–AtC3H3 and AtC3H3–sGFP constructs were strongly observed in the cytoplasm (Figure 4b), suggesting that AtC3H3 plays a role in the cytoplasm.

### 2.4. AtC3H3 Transcription Increases under Osmotic Stress Conditions

**Figure 5:**
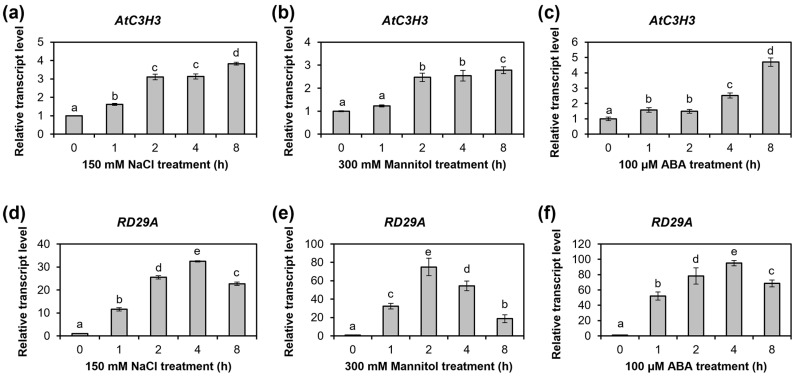
*AtC3H3* and *RD29A* expression patterns under osmotic stress conditions. (**a**–**c**) Relative *AtC3H3* transcript levels in 10 DAG WT seedlings treated with NaCl (**a**), mannitol (**b**), and ABA (**c**) for indicated times. (**d**–**f**) Relative *RD29A* transcript levels in 10 DAG WT seedlings treated with NaCl (**d**), mannitol (**e**), and ABA (**f**) for indicated times. *GAPc* was used to normalize the relative transcript levels. *AtC3H3* or *RD29A* transcript levels at 0 h of treatment were designated as 1. Data represent the average with standard deviations indicated by error bars (*n* = 6). Statistical differences (*p* < 0.05) are denoted by different letters.

To analyze *AtC3H3* expression under osmotic stress conditions, we assessed *AtC3H3* transcript levels using qRT-PCR in WT seedlings subjected to 150 mM NaCl, 300 mM mannitol, or 100 μM ABA for 0–8 h (Figure 5). *AtC3H3* transcript levels increased after treatment with NaCl, mannitol, and ABA for 1 h (Figure 5a–c). *RD29A* transcript levels were analyzed to validate proper treatments with NaCl, mannitol, and ABA (Figure 5d–f). We observed comparable results in semi-qRT-PCR analysis, with *AtC3H3* transcript levels increasing after NaCl, mannitol, and ABA treatments (Figure S2). These results indicate that *AtC3H3* may play a significant role in responding to osmotic stress. We thus focused on characterizing the function of *AtC3H3* in the osmotic stress response.

### 2.5. AtC3H3-Overexpressing Transgenic Plants Show Tolerance to Salt Stress

**Figure 6:**
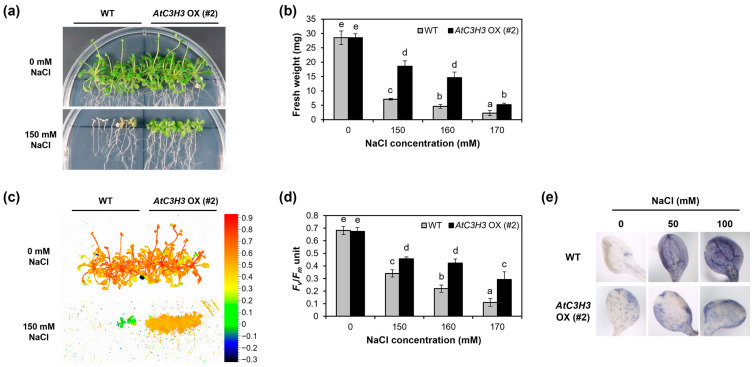
Response of *AtC3H3*-overexpressing transgenic plant (*AtC3H3* OX) seedlings to salt stress. (**a**) Photographs of WT and *AtC3H3* OX seedlings incubated under indicated NaCl concentrations for 23 days. (**b**) Fresh weight (FW) of WT and *AtC3H3* OX seedlings assessed after 23-day NaCl treatment. (**c**) Fluorescent image of photosystem II (PS II) activity (*F_v_*/*F_m_*) of WT and *AtC3H3* OX seedlings incubated under indicated NaCl concentrations for 23 days. (**d**) *F_v_*/*F_m_* units of WT and *AtC3H3* OX seedlings assessed after 23-day NaCl treatment. (**e**) Superoxide accumulation in cotyledons of 10 DAG WT and *AtC3H3* OX seedlings. Histochemical nitro blue tetrazolium staining carried out after NaCl treatment with indicated concentrations. In (**b**,**d**), data represent the average with standard deviations indicated by error bars (*n* = 24). Statistical differences (*p* < 0.05) are denoted by different letters.

To reveal the biological functions of *AtC3H3* in the osmotic stress response at the seedling stage, we generated *AtC3H3*-overexpressing transgenic plants (*AtC3H3* OXs) and selected three independent T_1_ lines using qRT-PCR and semi-qRT-PCR (Figure S3). T_3_ homozygous plants of the selected three T_1_ lines were isolated and used for subsequent experiments. To analyze the roles of *AtC3H3* in the salt stress response, *AtC3H3* OX seedlings were treated with various NaCl concentrations. Our results revealed that *AtC3H3* OXs were more tolerant with higher fresh weight (FW) than WT under 150, 160, and 170 mM NaCl treatments (Figure 6a,b, Figures S4a,b and S5a,b). Photosystem II (PS II) activity is often used to study plant physiology under salt stress conditions [22]. We assessed *F_v_*/*F_m_* representing PS II activity in both *AtC3H3* OXs and WT. *F_v_*/*F_m_* values were significantly higher in *AtC3H3* OXs than in WT under 150, 160, and 170 mM NaCl treatments (Figure 6c,d, Figures S4c,d and S5c,d). Abiotic stresses enhance reactive oxygen species production and accumulation in plant cells [23]. Consequently, we measured superoxide production in *AtC3H3* OXs and WT. After treatment with 50 and 100 mM NaCl, significantly less superoxide was accumulated in *AtC3H3* OXs than in WT (Figure 6e). These results demonstrate that *AtC3H3* OX seedlings show more tolerance to salt stress than WT seedlings. Next, using mannitol treatment, we examined how *AtC3H3* OXs respond to drought stress, which is another osmotic stress. We discovered no detectable differences in FW and PS II activity between *AtC3H3* OX and WT seedlings under 400, 450, and 500 mM mannitol treatments (Figure S6). These results indicate that *AtC3H3* OXs show no significant tolerance to drought than WT.

**Figure 7:**
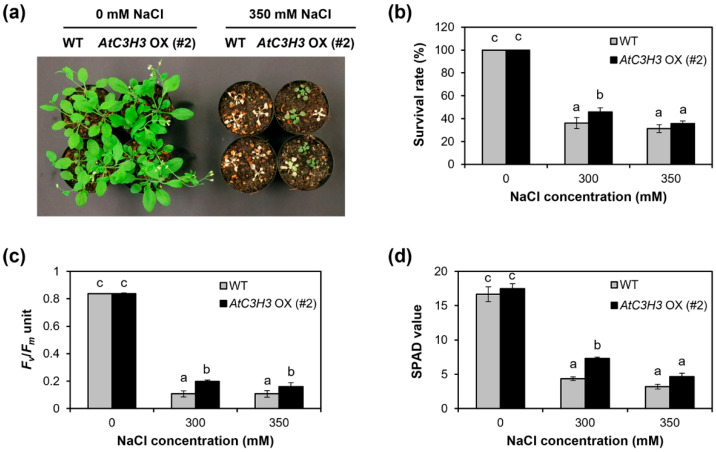
Response of mature *AtC3H3* OX plants to salt stress. (**a**) Photograph of WT and *AtC3H3* OXs treated with indicated NaCl concentrations for 17 days. (**b**) Survival ratio of WT and *AtC3H3* OXs after 17-day NaCl treatment. (**c**) *F_v_*/*F_m_* units of WT and *AtC3H3* OXs after 17-day NaCl treatment. (**d**) SPAD values of WT and *AtC3H3* OXs after 17-day NaCl treatment. In (**b**–**d**), data represent the average with standard deviations indicated by error bars (*n* = 15). Statistical differences (*p* < 0.05) are denoted by different letters.

To study the response of mature *AtC3H3* OXs to salt stress, we exposed *AtC3H3* OXs and WT grown on soil with 0, 300, and 350 mM NaCl. The results showed that *AtC3H3* OXs were more tolerant than WT under 300 and 350 mM NaCl treatments (Figure 7a,b and Figure S7a,b). Since chlorophyll content is crucial for estimating the photosynthesis capacity of plants [22], chlorophyll content as well as PS II activity were quantified in *AtC3H3* OXs and WT. Notably, PS II activity, as indicated by the *F_v_*/*F_m_* value, was higher in *AtC3H3* OXs than in WT under the NaCl treatments (Figure 7c and Figure S7c). Additionally, *AtC3H3* OXs displayed greater chlorophyll contents, as indicated by the SPAD value, than WT under the NaCl treatments (Figure 7d and Figure S7d). These and previous results suggest that *AtC3H3* OXs are more tolerant to salt stress than WT at the seedling and mature plant stages. Furthermore, to confirm the response of mature *AtC3H3* OXs to drought stress, we subjected them to drought stress. We found no significant differences in PS II activity and chlorophyll content between *AtC3H3* OXs and WT under drought treatments (Figure S9). Our results suggest that *AtC3H3* overexpression enhances tolerance, specifically to salt stress, in both seedlings and mature plants.

### 2.6. AtC3H3 OXs Show the Elevated Expression of Both ABA-Dependent and -Independent Salt Stress-Responsive Genes

**Figure 8:**
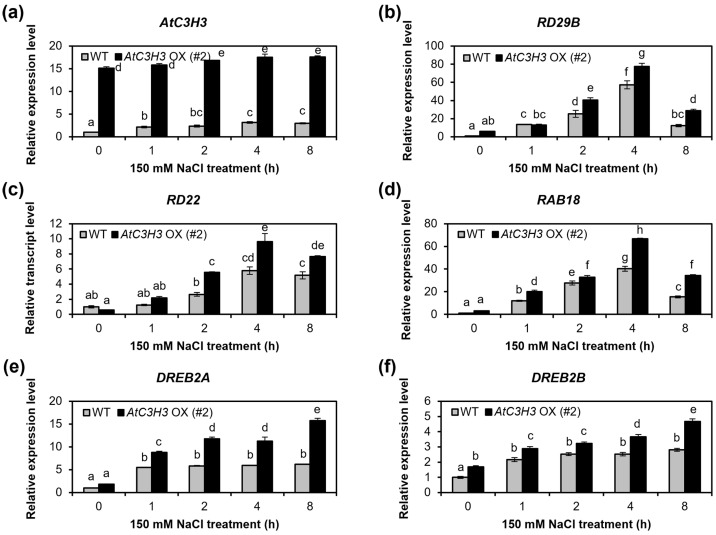
Representative ABA-dependent and -independent salt stress-responsive gene expression patterns in *AtC3H3* OXs. Relative *AtC3H3* (**a**), *RD29B* (**b**), *RD22* (**c**), *RAB18* (**d**), *DREB2A* (**e**), and *DREB2B* (**f**) transcript levels in WT and *AtC3H3* OX seedlings treated with NaCl for indicated times. *GAPc* was used to normalize the relative transcript levels. Transcript levels of each gene in WT at 0 h of NaCl treatment were set to 1. Data represent the average with standard deviations indicated by error bars (*n* = 6). Statistical differences (*p* < 0.05) are denoted by different letters.

The response of plants to salt stress is influenced by ABA-dependent and/or -independent signaling pathways [2,3]. To investigate the *AtC3H3*-mediated salt stress signaling pathway, we analyzed the expression patterns of ABA-dependent and -independent stress-related genes under salt stress conditions in *AtC3H3* OXs and WT. The results of qRT-PCR indicated that the transcript levels of all examined genes were elevated after NaCl treatment (Figure 8 and Figure S9). The transcript levels of the ABA-dependent stress-responsive genes *RD29B*, *RD22*, and *RAB18* were significantly higher in *AtC3H3* OXs than in WT under the NaCl treatments (Figure 8b–d and Figure S9b–d). Interestingly, *DREB2A* and *DREB2B*, representative ABA-independent stress-responsive genes, also showed significantly higher transcript levels in *AtC3H3* OXs than in WT under NaCl treatment conditions (Figure 8e,f and Figure S9e,f). These results suggest that the *AtC3H3*-mediated response to salt stress may occur through both ABA-dependent and -independent signaling pathways.

### 2.7. Analysis of Target mRNAs of the RNase Function of AtC3H3 Using mRNA-Sequencing

In a previous study, AtC3H3 was identified as an RNase [21]. To identify the target mRNAs of AtC3H3 RNase function and stress-responsive genes involved in salt tolerance in *AtC3H3* OXs, we performed mRNA sequencing (mRNA-Seq) analysis using *AtC3H3* OXs and WT under the NaCl treatments. mRNA-Seq reads were successfully mapped to the *Arabidopsis* Columbia genome, with 95.7–96.1% mapping rates (Table S1). The number of mapped reads ranged from 21.1 to 27.2 million (Table S1). We excluded less abundant genes, leaving us with 37,982 genes for the analysis.

Genes with more than 2-fold differences in expression, along with *p* < 0.05, were considered up- or down-regulated in *AtC3H3* OXs. The results indicated that only 10 and 11 genes were up- and down-regulated in *AtC3H3* OXs, respectively (Table 1, Tables S2 and S3). We examined the gene ontology (GO) enrichment of the up- and down-regulated genes to understand the *AtC3H3*-associated biological processes. The up-regulated genes showed enrichment in response to insects, glycosinolate biosynthetic process, S-glycoside biosynthetic process, glucosinolate biosynthetic process, and glycosyl compound biosynthetic process. The down-regulated genes were enriched in the secondary metabolic process (Table S4).

We hypothesized that the target mRNAs of the RNase function of AtC3H3 would be down-regulated in *AtC3H3* OXs. In addition, we expected that salt stress-responsive genes involved in salt tolerance would be down-regulated in *AtC3H3* OXs under salt stress conditions because *AtC3H3* expression increased under salt stress conditions. We analyzed the expression of the 11 down-regulated genes using Genevestigator (Table S2). Among them, only six, including *Dark Inducible 10* (*DIN10*)/*Raffinose Synthase 6* (*RS6*), AT5G04790, *Cinnayl-Alcohol Dehydrogenase 8* (*CAD8*)/*Elicitor-activated gene 3-2* (*ELI3-2*), *ATP Responsive 2* (*ATPR2*)/*Farnesoic Acid Methyl Transferase-Like* (*FAMT-L*), *DIN11*, and *Isocitrate Lyase* (*ICL*), were available in the Genevestigator database for analysis. Unexpectedly, the expression of the six genes increased under salt, drought, and osmotic stress conditions in Genevestigator analysis (Figure S10). These results suggest that AtC3H3 might not target the down-regulated genes from the mRNA-Seq analysis and might potentially target noncoding RNAs, including long noncoding RNAs and microRNAs (miRNAs), but not mRNAs.

## 3. Discussion

### 3.1. AtC3H3 OXs Show Salt Tolerance but Not Drought Tolerance

Upon detecting stress, plants generate second messengers that activate a series of downstream genes. The CCCH zinc-finger proteins then respond to environmental stresses by influencing gene expression at the transcriptional and post-transcriptional levels through various signaling pathways [6]. This study investigated the molecular and biological functions of *AtC3H3*, a non-TZF gene, in *Arabidopsis*. Our analysis demonstrates that *AtC3H3* is involved in the salt stress response. *AtC3H3* expression was elevated under NaCl, mannitol, and ABA treatments (Figure 5 and Figure S2). Furthermore, *AtC3H3* OXs presented greater tolerance to salt stress than WT at both the seedling and mature plant stages (Figure 6, Figure 7, Figures S4, S5 and S7), indicating a significant role for *AtC3H3* in salt stress tolerance in *Arabidopsis*. Interestingly, although *AtC3H3* expression was elevated under mannitol treatment, *AtC3H3* OXs did not show significant differences compared with WT in drought response (Figure 5, Figures S2, S6 and S8), indicating that *AtC3H3* may not play an important role in drought tolerance in *Arabidopsis*.

### 3.2. AtC3H3 Enhances Salt Tolerance by Increasing the Expression of Both ABA-Dependent and -Independent Salt Stress-Responsive Genes

Many CCCH zinc-finger proteins participate in stress responses through various mechanisms. Some CCCH zinc-finger proteins enhance stress tolerance through the ABA signaling pathway in plants. Single mutants *attzf10*/*atszf2-1* and *attzf11*/*atszf1-1* exhibited significantly increased expression of ABA-dependent stress-related genes, including *Cold-Regulated 15A* (*COR15A*), *COR47*, *RD29A*, and *KIN1* [7]. *AtC3H17* OXs exhibited higher expression of ABA-dependent salt stress-related genes such as *RAB18*, *COR15A*, and *RD22*, whereas *atc3h17* mutants showed lower expression [10]. *IbC3H18*, a non-TZF protein gene in sweet potato, is also responsible for ABA-signaling-mediated tolerance to salt stress [14]. In contrast, some CCCH zinc-finger proteins are involved in the stress response through ABA-independent pathways. For example, *OsC3H10* contains both DREs and ABREs in its promoter region. Notably, DREs are *cis*-elements regulated by the drought-induced OsDREB2 transcription factor, suggesting that drought may induce *OsC3H10* expression in an ABA-independent manner [24].

In this study, we analyzed the expression of well-known ABA-dependent and -independent salt stress-responsive genes in WT and *AtC3H3* OXs under salt stress conditions. The qRT-PCR results demonstrated higher expression of ABA-dependent salt stress-responsive genes, namely *RD29B*, *RD22*, and *RAB18*, and ABA-independent salt stress-responsive genes, namely *DREB2A* and *DREB2B*, in *AtC3H3* OXs than in WT under salt stress treatment (Figure 8 and Figure S9). This provides a new perspective on the role of *AtC3H3* in mediating the salt stress response. The novelty of our findings lies in identifying the *AtC3H3*-mediated salt stress response signal transduction. Similarly, the involvement of *PvC3H72* in cold stress tolerance is mediated by regulating the Inducer of CBF Expression 1 (ICE1)–CBF–COR regulon as well as ABA-related genes in switchgrass (*Panicum virgatum*). It plays a role in the cold stress response via both ABA-dependent and -independent pathways [25]. These findings expand our knowledge of how plants respond to stress.

### 3.3. Targets of the RNase Function of AtC3H3

Some CCCH zinc-finger proteins play a role in post-transcriptional regulation by modulating RNA metabolism in plants under stress. In an early study of CCCH zinc-finger proteins, Tristetraprolin (TTP) and Butyrate Response Factor 1 (BRF1) were discovered in humans before being found in plants. These proteins bind to AU-rich elements in the 3′ UTR of mRNA and regulate mRNA turnover [26,27]. Most CCCH zinc-finger proteins studied early in plants, such as TZFs, are involved in RNA binding and regulation at post-transcriptional stages. The arginine-rich (RR) site of plant-specific RR-TZF plays a crucial role in RNA binding. The CCCH zinc-finger proteins localize to stress granules (SGs) and processing bodies (PBs). They initiate RNA degradation in the PBs and regulate stress responses as well as plant development by binding to specific RNA elements in the SGs [28]. Furthermore, certain proteins, including AtTZF1, AtTZF4/SOM, AtTZF5, AtTZF6/PEI1, OsTZF1, and OsC3H10, shuttle between the nucleus and cytoplasm and co-localize with SGs and PBs [24,29]. For example, AtTZF1 and OsTZF1 enhance abiotic stress tolerance by regulating stress-related genes, probably through RNA metabolism [24,29].

AtC3H3 binds to RNAs and acts as an RNase in vitro [21]. Additionally, AtC3H3 was localized in the cytoplasm (Figure 4), suggesting its probable involvement in RNA metabolism. To identify the target mRNAs of AtC3H3, mRNA-Seq was performed to compare gene expression in WT and *AtC3H3* OXs under normal and salt stress conditions. We could not identify any potential target mRNAs from the mRNA-Seq analysis, indicating that AtC3H3 may be involved in the metabolism of RNAs other than mRNAs via its RNase activity. Indeed, Roquin 1, a CCCH zinc-finger protein, has recently been identified as a critical miRNA homeostasis regulator in humans. Roquin 1 decreases the half-life of mature miRNAs by increasing their mono-uridylation [30]. Similarly, monocyte chemotactic protein-induced protein 1 (MCPIP1), a CCCH zinc-finger protein and RNase, interacts with GW182, a core component of miRNA-induced silencing complex. Notably, MCPIP1 also suppresses miRNA biogenesis by counteracting Dicer, a central RNase involved in miRNA processing [31]. In *Arabidopsis*, AtC3H15/AtCDM1, a human TTP ortholog, is involved in pollen wall pattern formation by regulating miRNA maturation [32]. Our mRNA-Seq analysis suggested that noncoding RNAs, such as miRNAs or long noncoding RNAs, may be targets of AtC3H3 as an RNase. Further studies, including noncoding RNA-Seq, are necessary to identify AtC3H3 targets.

## 4. Materials and Methods

### 4.1. Arabidopsis Growth

The *Arabidopsis* plants in this study belonged to the Columbia (Col-0) ecotype. Seed preparation, germination, and plant growth followed the previous procedures [33].

### 4.2. Multiple Sequence Alignment

NCBI BLAST was used for BLASTP analysis (https://blast.ncbi.nlm.nih.gov/Blast.cgi, accessed on 24 March 2021). In the BLASTP analysis, the database was set to reference proteins (ref_seq protein) and other parameters were set to default.

Clustal Omega Multiple Sequence Alignment was used for the multiple sequence alignments (https://ebi.ac.uk/jdispatcher/msa/clustalo, accessed on 24 March 2021). In the alignments, protein sequences were input in FASTA format. The output format was set to ClustalW, and other parameters were set to default. GeneDoc (https://nrbsc.org/gfx/genedoc, accessed on 24 March 2021) was used to visualize the results of the multiple sequence alignment.

### 4.3. Vector Construction

To construct sGFP-fused vectors used for analyzing subcellular localization, the full-length *AtC3H3* open reading frame (ORF) was inserted into pFGL1283 and pFGL1292 in frames with N-terminal and C-terminal sGFP, respectively, under the control of a modified CaMV *35S* promoter [33].

To construct a *GUS*-fused vector for the histochemical GUS assay, a 2-kb upstream region from the transcription initiation site of *AtC3H3*, including a 185-bp 5′ UTR, was isolated as the *AtC3H3* promoter and fused to the *GUS* gene [33].

To construct a vector for *AtC3H3* overexpression, the full-length *AtC3H3* ORF was inserted into pFGL1434, containing the modified CaMV *35S* promoter to control *AtC3H3* expression and an N-terminal-fused hemagglutinin tag [33].

The primers used for cloning in this study are listed in Table S5.

### 4.4. Transgenic Plants Generation

The binary vectors were introduced into *Agrobacterium tumefaciens* GV3101 (pMP90) using the freeze–thaw method and transferred into WT *Arabidopsis* using the floral-dipping technique [34,35]. Kanamycin (50 μg/mL) was used to isolate transgenic *Arabidopsis* plants. T_3_ homozygous lines were selected for subsequent experiments.

### 4.5. Stress Treatment

Prior to conducting RT-PCR analyses, 10 days after germination (DAG) seedlings grown under short-day (SD) conditions were subjected to stress treatments with an MS solution containing 150 mM NaCl, 300 mM mannitol, or 100 μM ABA for 0, 1, 2, 4, and 8 h, after which they were collected.

For determining the salt and drought stress responses, 5 DAG seedlings grown under SD conditions were transplanted into MS agar media supplemented with 0, 150, 160, or 170 mM NaCl or 0, 400, 450, or 500 mM mannitol for 7, 18, or 23 days.

For determining the salt stress response of mature plants, 21 DAG plants grown under long-day (LD) conditions were irrigated with 0, 300, and 350 mM NaCl at 3- or 4-day intervals.

For determining the drought stress response of mature plants, 3-week-old plants grown under LD conditions were deprived of water for 17 days and then rewatered for 5 days.

### 4.6. Histochemical Staining for Detection of Superoxide Production

For superoxide staining for histochemical analysis, 10 DAG seedlings grown under SD conditions were subjected to filter paper soaked in 0, 50, or 100 mM NaCl for 2 h. Subsequently, the seedlings were stained with a nitro blue tetrazolium solution following the previous description [10].

### 4.7. F_v_/F_m_ and SPAD Value Measurement

FluorCam FC-800 (Photon Systems Instruments, Drasov, Czech Republic) and Jun-ior-PAM (Heinz Walz GmbH, Effeltrich, Germany) were used for *F_v_*/*F_m_* unit determination in seedlings and the third or fourth rosette leaves of mature plants, respectively. The SPAD-502 plus chlorophyll meter (Konica Minolta, Inc., Tokyo, Japan) was used for SPAD value determination in the third or fourth rosette leaves of mature plants. The *F_v_*/*F_m_* unit and SPAD value were determined in accordance with the manufacturer’s instructions.

### 4.8. RNA Isolation and RT-PCR

The RNAqueous RNA Isolation Kit (Invitrogen, Carlsbad, CA, USA) and Plant RNA Isolation Aid (Invitrogen) were used for total RNA isolation as described in the manufacturer’s instructions. Reverse transcription using total RNA was conducted as previously described [10].

qRT-PCR was conducted using Power SYBR^TM^ Green PCR Master Mix (Applied Biosystems, Foster City, CA, USA) on the QuantStudio^TM^ 3 real-time PCR system (Applied Biosystems) following the manufacturer’s instructions. Real-time DNA amplification was analyzed using the QuantStudio^TM^ Design and Analysis software v.1.4.3 (Applied Biosystems). Two technical replicates were used for each biological replicate. Three independent reactions were performed for each technical replicate.

Semi-qRT-PCR was performed as previously described [10]. Notably, PCR was repeated for 30–31 cycles for *AtC3H3* and 23–24 cycles for *GAPc* and *RD29A*.

The primers used for RT-PCR in this study are listed in Table S6.

### 4.9. Histochemical GUS Assay

GUS staining for the histochemical assay was carried out as previously described [33]. Briefly, the seedlings were incubated in a solution containing 2 mM 5-bromo-4-chloro-3-indolyl-β-D-glucuronic acid, 50 mM phosphate buffer (pH 7.0), 0.5 mM potassium ferricyanide, and 0.5 mM potassium ferrocyanide at 37 °C in the dark for 6 h. Then, the seedlings were washed with 50 mM phosphate buffer (pH 7.0) and soaked in a solution of 100% ethanol and acetic acid (9:1, *v*/*v*) overnight at room temperature for fixation and clearing.

### 4.10. Protoplast Transformation

Protoplasts from *Arabidopsis* were isolated and transformed using polyethylene glycol, as described previously [36].

### 4.11. Statistical Analysis

The IBM SPSS Statistics software version v.27.0.0.0 (IBM Corp., Armonk, NY, USA) was used for statistical analysis. Statistical differences were measured using one-way ANOVA with Tukey’s multiple comparison test subsequent to the Shapiro–Wilk normality test.

### 4.12. Library Preparation and mRNA-Seq

Ten-day-old WT and *AtC3H3* OX seedlings were exposed to 150 mM NaCl for 1, 2, or 4 h and then total RNA was isolated. Two micrograms of total RNA from each treatment condition were combined and used to prepare an mRNA-Seq library, as described previously [37]. The HiSeq X10 system (Illumina, Inc., San Diego, CA, USA) was used for high-throughput paired-end 100 bp sequencing. The mRNA-Seq library was prepared and sequenced by E-biogen (https://www.e-biogen.com, accessed on 29 April 2022). Two biological replicates were used for each sample.

### 4.13. mRNA-Seq

mRNA-Seq data was analyzed by E-biogen (https://www.e-biogen.com, accessed on 29 April 2022). The raw sequencing data underwent quality control using FastQC [38]. FASTX_Trimmer and BBMap were used to remove low-quality reads (<Q20) and adapters [39,40]. TopHat was used to map the trimmed reads to the *Arabidopsis* genome sequence TAIR 10 as the reference genome [41]. The fragments per kb per million reads (FPKM) values were used to estimate gene expression levels as determined by Cufflinks [42]. The FPKM values were normalized using EdgeR based on the quantile normalization method [43]. The RNA-Seq mapping rates ranged from 95.7% to 96.1%. The mapped reads ranged from 21.1 to 27.2 million (Table S1). Alignment rates ranged from 95.7% to 96.1% (Table S1). ExDEGA (E-biogen, Inc., Seoul, Republic of Korea) was used for data mining and graphic visualization. The complete mRNA-Seq data from this study is available on the Gene Expression Omnibus database (http://www.ncbi.nlm.nih.gov/geo, accessed on 28 August 2024) under accession number GSE275862. DAVID (https://david.ncifcrf.gov/, accessed on 19 September 2022) with default parameters was used to analyze GO annotation enrichment.

## 5. Conclusions

In this study, we studied the role of *AtC3H3*, a non-TZF gene, in the response of *Arabidopsis* to salt stress. Our findings demonstrate that *AtC3H3* expression increases under osmotic stress, such as high salinity and drought stress. *AtC3H3*-overexpressing *Arabidopsis* seedlings exhibit improved salt tolerance. Furthermore, *AtC3H3* plays a role in the salt stress response of *Arabidopsis* by influencing both ABA-dependent and -independent pathways. These findings not only deepen our knowledge of plant stress responses but also hold promise for developing new strategies to enhance crop tolerance in the face of increasing salinity.

## Figures and Tables

**Table 1:** Number of differentially expressed genes (DEGs).

Experiment	Up-Regulated Gene Number	Down-Regulated Gene Number
*AtC3H3* OX NaCl vs WT NaCl	10	11

## Data Availability

The data presented in this study are available in the Supplementary Materials. The data presented in this study are openly available in Gene Expression Omnibus (GEO); the GEO accession number is GSE275862.

## Supplementary Materials

The following supporting information can be downloaded at https://www.mdpi.com/article/10.3390/ijms252010943/s1.
