# Supplementary material for: AtC3H3, an Arabidopsis Non-TZF Gene, Enhances Salt Tolerance by Increasing the Expression of Both ABA-Dependent and -Independent Stress-Responsive Genes
Source: Int J Mol Sci. 2024 Oct 11;25(20):10943. doi: 10.3390/ijms252010943 (PMC11507560; doi:10.3390/ijms252010943)
Supplement: Supplementary file 1 [file ijms-25-10943-s001.zip › ijms-3236518-supplementary.pdf]

Supplementary data

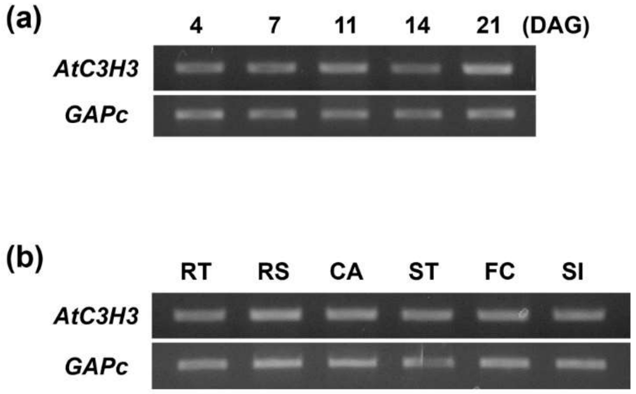

Figure S1. Temporal and spatial expression of *AtC3H3*. (**a**) Semi-qRT-PCR analysis of *AtC3H3* in 4-, 7-, 11-, 14-, and 21-day-old WT seedlings grown under SD conditions. (**b**) Semi-qRT-PCR analysis of *AtC3H3* expression in organs of 49-day-old WT grown under LD conditions. RT, roots; RS, rosette leaves; CA, cauline leaves; ST, stems; FC, floral clusters; SI, siliques. *GAPc* was used for an internal control. At least two biological replicates showed similar results, with one shown here.

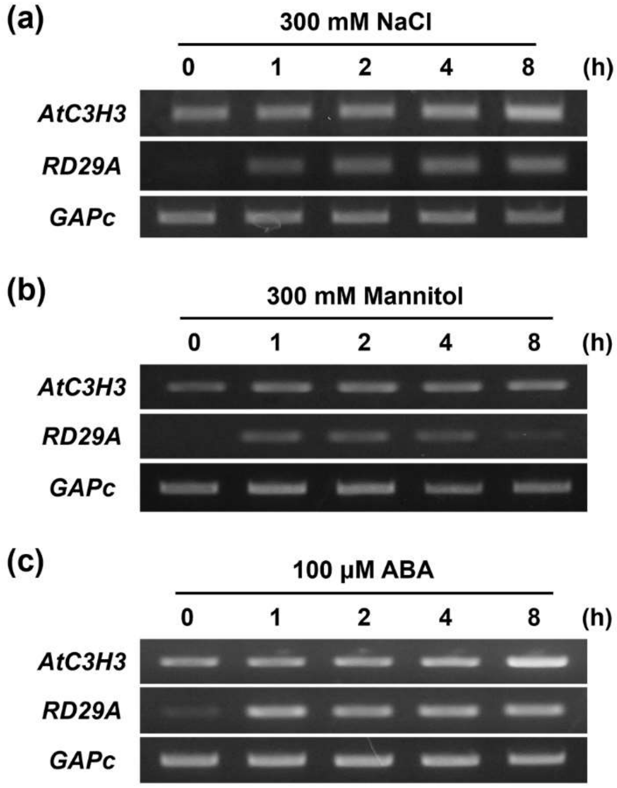

Figure S2. Expression analysis of *AtC3H3* under osmotic stress conditions. (**a**) Expression of *AtC3H3* and *RD29A* under 300 mM NaCl treatment for 0, 1, 2, 4, and 8 h. (**b**) Expression of *AtC3H3* and *RD29A* under 300 mM mannitol treatment for 0, 1, 2, 4, and 8 h. **c** Expression of *AtC3H3* and *RD29A* under 100 μM ABA treatment for 0, 1, 2, 4, and 8 h. *GAPc* was used as an internal control. At least two biological replicates showed similar results, with one shown here.

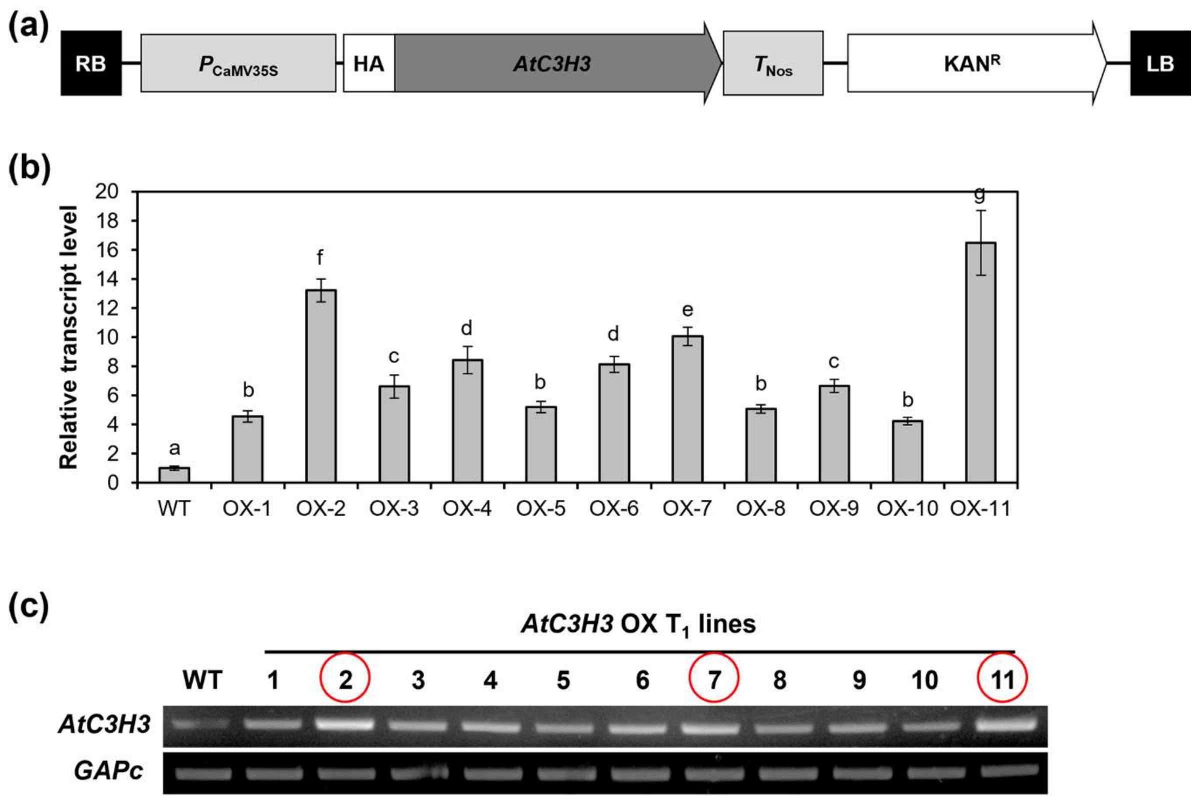

Figure S3. Isolation of *AtC3H3* OXs. (**a**) Schematic map of vector for *AtC3H3* overexpression. (**b**) Selection of *AtC3H3*-overexpressing T_1 lines by qRT-PCR. Transcript level in WT was set as 1. Three independent reactions were performed for each technical replicate. Two technical replicates were performed for each biological replicate. Data shown are the mean ± S.D. ($n = 6$). At least two biological replicates showed similar results, with one shown here. Different letters display significant differences ($p < 0.05$). (**c**) Selection of T_1 lines overexpressing *AtC3H3* by semi-qRT-PCR. Circled lines were selected for further analysis.

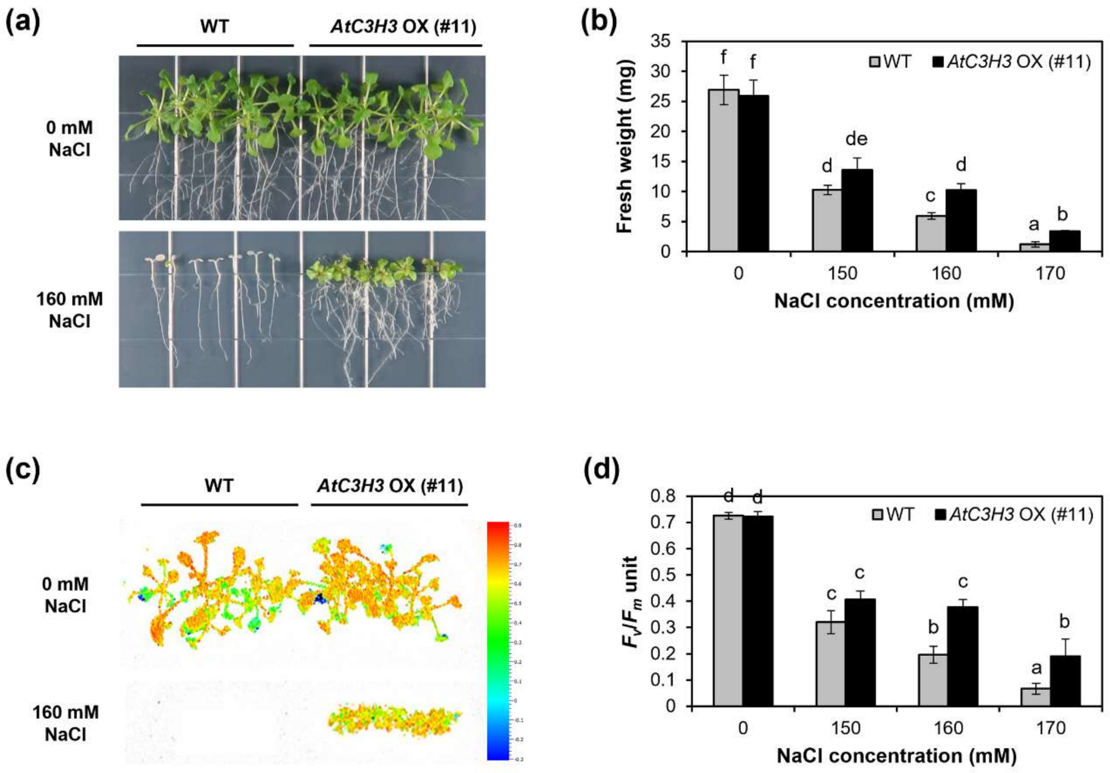

Figure S4. Salt stress response of *AtC3H3* OX (#11) at seedlings stage. (**a**) Five-day-old WT and *AtC3H3* OX (#11) seedlings were transferred onto 0, 150, 160, and 170 mM NaCl-containing medium and incubated. Photographs were taken after 18-day NaCl treatment. (**b**) FW of WT and *AtC3H3* OX (#11) seedlings was measured after 18-day NaCl treatment. Data shown are the means ± S.D. (n = 24 plants). Different letters display significant differences ($p < 0.05$). (**c**) Fluorescent image of PS II activity (F_v/F_m) was taken after 18-day NaCl treatment. (**d**) PS II activity (F_v/F_m) was measured after 18-day NaCl treatment using FluorCam. Data shown are the means ± S.D. (n = 24 plants). Different letters display significant differences ($p < 0.05$).

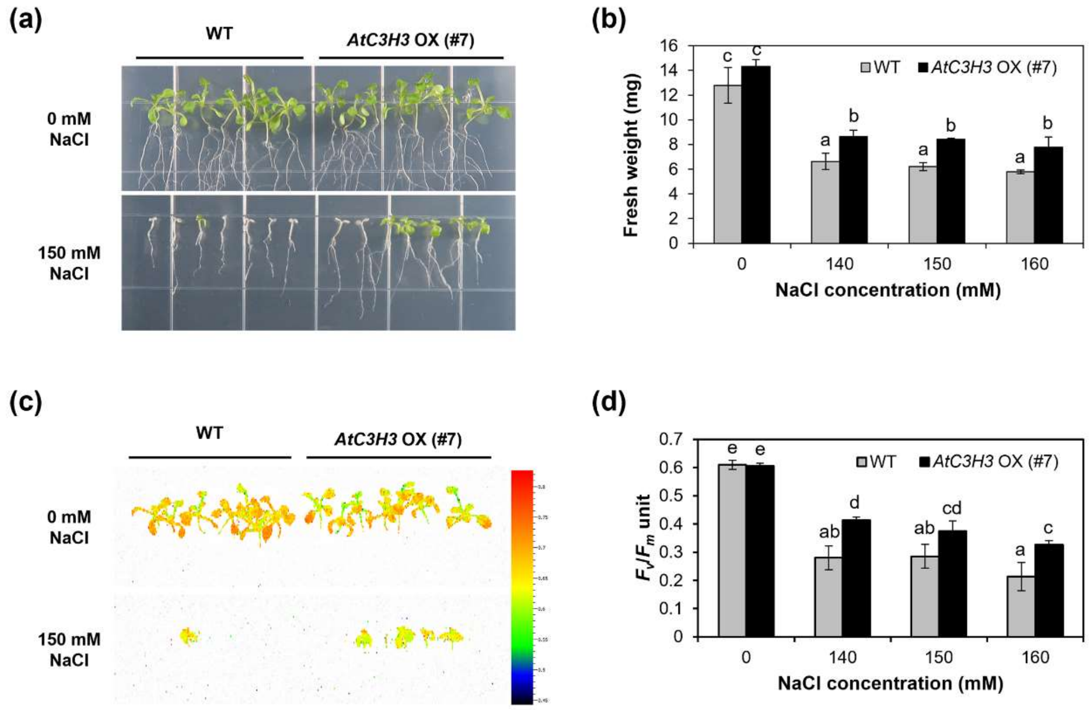

Figure S5. Salt stress response of *AtC3H3* OX (#7) at seedlings stage. (**a**) Five-day-old WT and *AtC3H3* OX (#7) seedlings were transferred onto 0, 140, 150, and 160 mM NaCl-containing medium and incubated. Photographs were taken after 12-day NaCl treatment. (**b**) FW of WT and *AtC3H3* OX (#7) seedlings was measured after 12-day NaCl treatment. Data shown are the means ± S.D. (n = 24 plants). Different letters display significant differences ($p < 0.05$). (**c**) Fluorescent image of PS II activity (F_v/F_m) was taken after 12-day NaCl treatment. (**d**) PS II activity (F_v/F_m) was measured after 12-day NaCl treatment using FluorCam. Data shown are the means ± S.D. (n = 24 plants). Different letters display significant differences ($p < 0.05$).

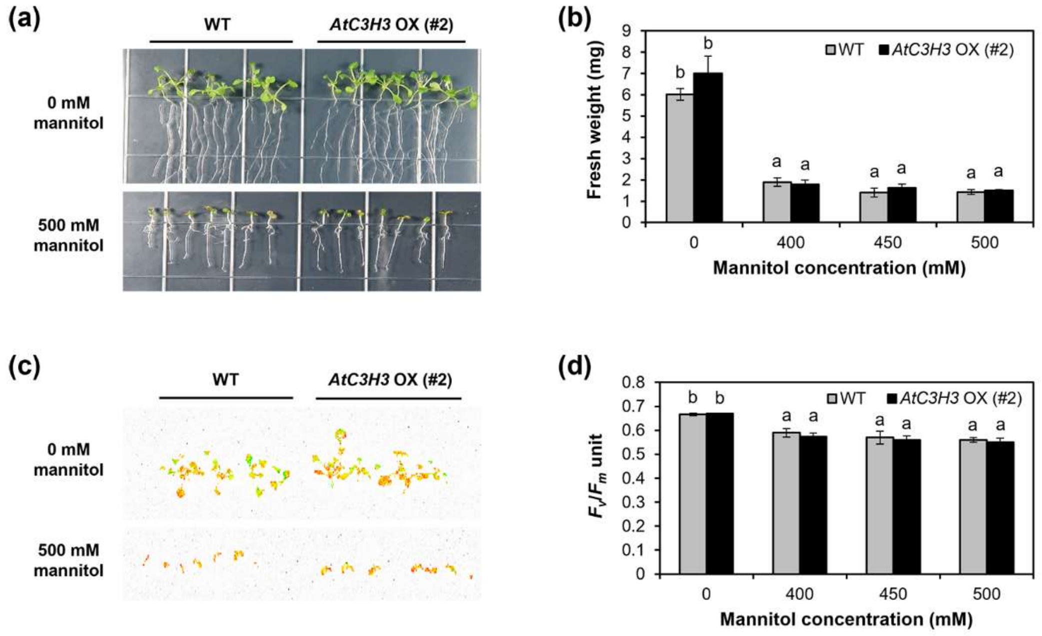

Figure S6. Drought stress response of *AtC3H3* OX at seedlings stage. (**a**) Five-day-old WT and *AtC3H3* OX seedlings were transferred onto 0, 400, 450, and 500 mM mannitol-containing medium and incubated. Photographs were taken after 7-day mannitol treatment. (**b**) FW of WT and *AtC3H3* OX seedlings was measured after 7-day mannitol treatment. Data shown are the means ± S.D. (n = 24 plants). Different letters display significant differences ($p < 0.05$). (**c**) Fluorescent image of PS II activity (F_v/F_m) was taken after 7-day mannitol treatment. (**d**) PS II activity (F_v/F_m) was measured after 7-day mannitol treatment using FluorCam. Data shown are the means ± S.D. (n = 24 plants). Different letters display significant differences ($p < 0.05$).

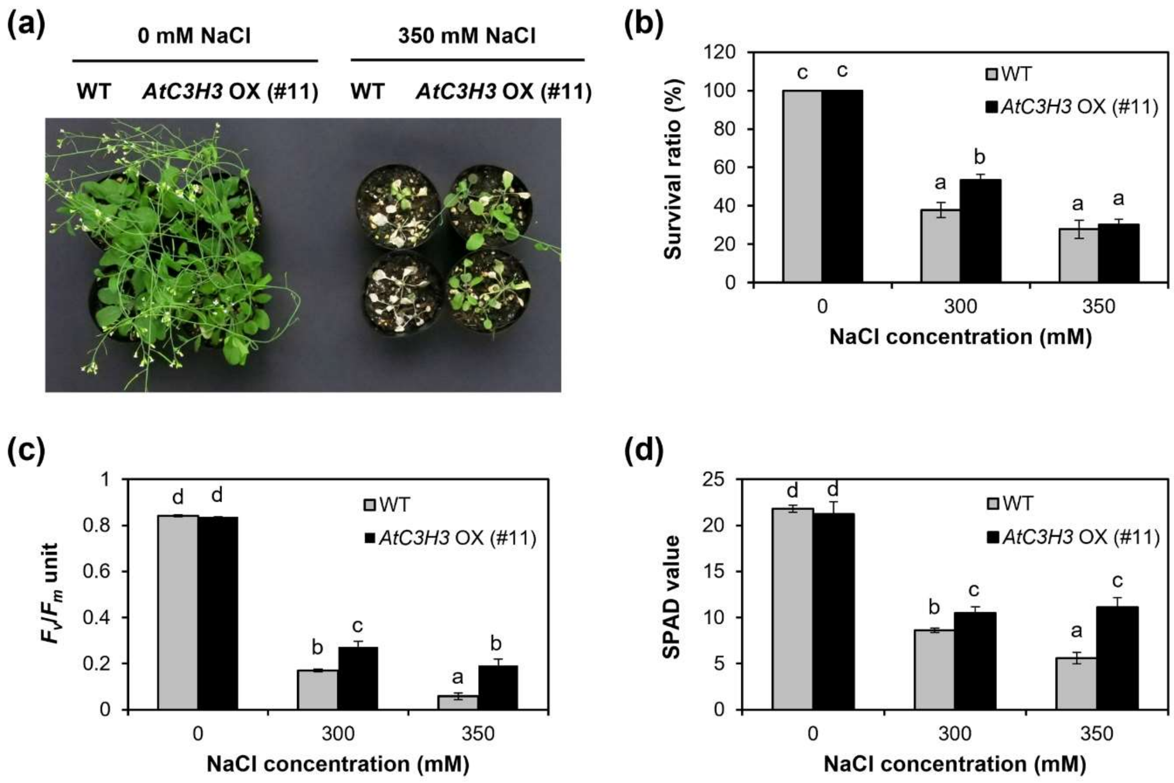

Figure S7. Response of *AtC3H3* OX (#11) mature plants to salt stress. (**a**) Photograph of WT and *AtC3H3* OX (#11) plants treated with indicated concentrations of NaCl for 17 days. (**b**) Survival ratio of WT and *AtC3H3* OXs (#11) after 17-day NaCl treatment. (**c**) F_v/F_m units of WT and *AtC3H3* OXs (#11) after 17-day NaCl treatment. (**d**) SPAD values of WT and *AtC3H3* OXs (#11) after 17-day NaCl treatment. In (**b**)–(**d**), the data represents the average with standard deviations indicated by error bars (n = 15 plants). Statistical differences ($p < 0.05$) are denoted by different letters.

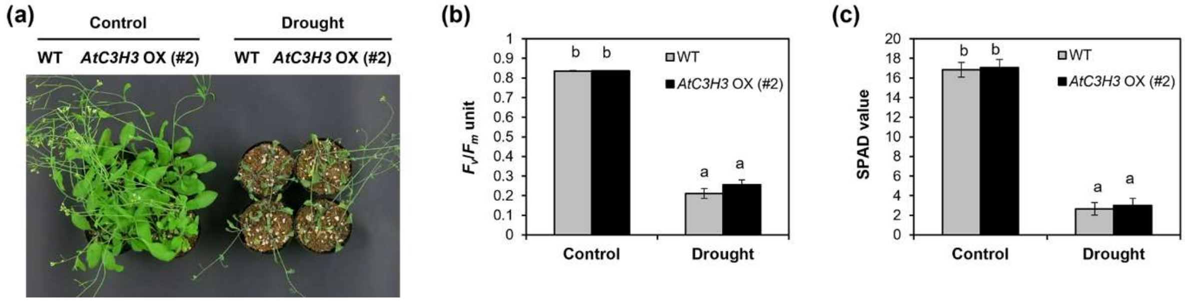

Figure S8. Drought-stress response of *AtC3H3* OX mature plants. (**a**) Three-week-old WT and *AtC3H3* OX plants were dried for 17 days and then rewatered. Photographs were taken after five days of rewatering. (**b**) PS II activity (F_v/F_m) of WT and *AtC3H3* OX dried for 17 days and rewatered for five days. (**c**) SPAD values of WT and *AtC3H3* OX dried for 17 days and rewatered for five days. Data shown are the means ± S.D. (n = 15 plants). Different letters display significant differences ($p < 0.05$).

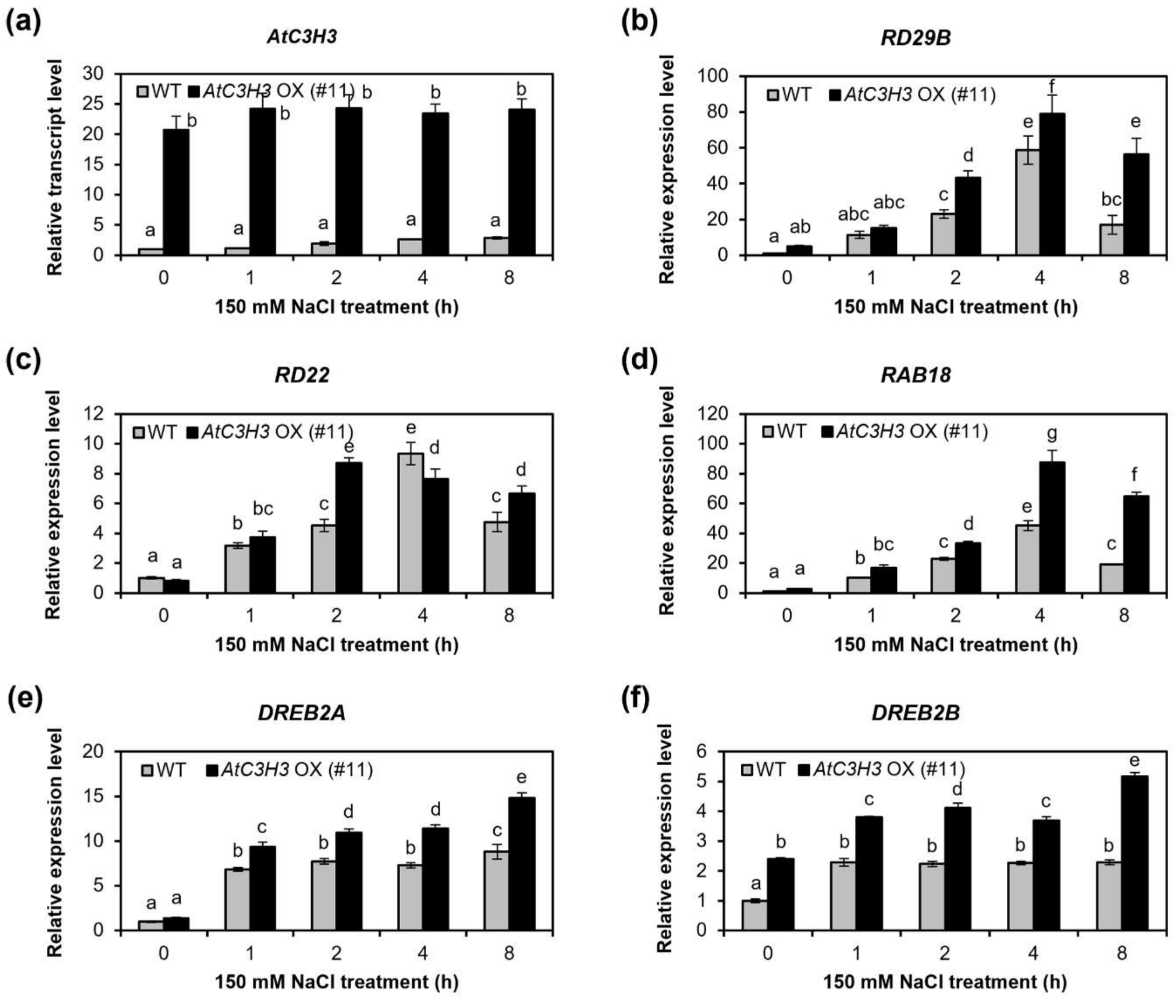

Figure S9. Expression patterns of representative ABA-dependent and -independent salt stress-responsive genes in *AtC3H3* OXs (#11). Relative transcript levels of *AtC3H3* (**a**), *RD29B* (**b**), *RD22* (**c**), *RAB18* (**d**), *DREB2A* (**e**), and *DREB2B* (**f**) in WT and *AtC3H3* OX (#11) seedlings treated with NaCl for indicated times. *GAPc* was used to normalize the relative transcript levels. Transcript levels of each gene in WT at 0 h of NaCl treatment were set to 1. The data represents the average with standard deviations indicated by error bars ($n = 6$). Statistical differences ($p < 0.05$) are denoted by different letters.

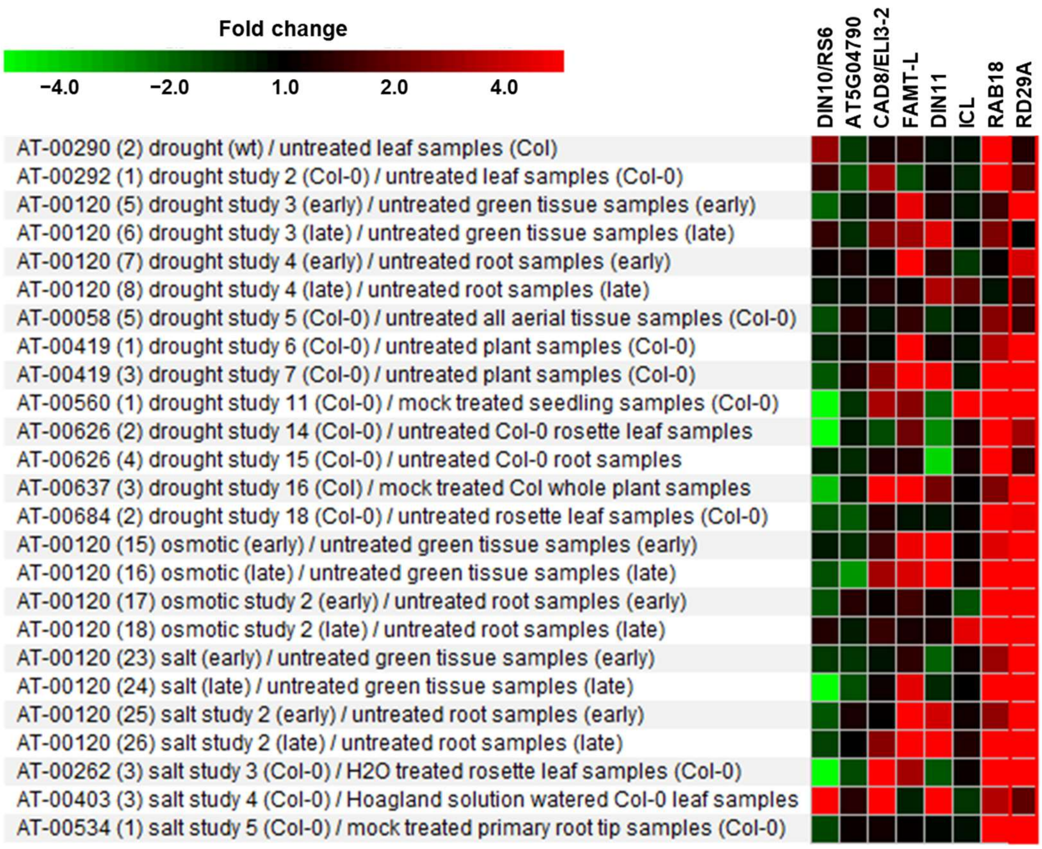

Figure S10. Expression analysis of 6 downregulated genes in *AtC3H3* OXs under drought, osmotic, and salt stress conditions using Genevestigator. *RD29A* and *RAB18* were used as marker genes for stress conditions.

Table S1. Suitability of RNA-Sequencing data

Sample	Forward reads		Reverse reads		Mapping rate	Aligned pairs	Pair alignment rate
	Input	Mapped	Input	Mapped			
WT-NaCl-1	24,555,255	24,098,795	24,555,255	23,923,936	97.8%	23,815,153	96.1%
WT-NaCl-2	27,168,480	26,618,921	27,168,480	26,418,597	97.6%	26,273,020	95.7%
AtC3H3 OX-NaCl-1	22,889,799	22,493,047	22,889,799	22,358,926	98.0%	22,263,847	96.0%
AtC3H3 OX-NaCl-2	21,568,345	21,190,760	21,568,345	21,071,941	98.0%	20,971,908	96.1%

Table S2. List of up-regulated genes in *AtC3H3* OX under salt stress condition

Locus tag	Gene Symbol	Fold change	*p*-value	Description
AT5G13565	AT5G13565	17.217	0.000	Hypothetical protein
AT5G20240	PI	12.424	0.001	Floral homeotic gene encoding a MADS domain transcription factor
AT4G18203	AT4G18203	3.322	0.045	Hypothetical protein
AT1G76465	AT1G76465	2.712	0.000	Hypothetical protein
AT5G25756	AT5G25756	2.485	0.012	Hypothetical protein
AT5G65080	MAF5/AGL68	2.477	0.031	MADS-domain protein
AT3G26135	AT3G26135	2.281	0.038	2-oxoglutarate/Fe(II)-dependent oxygenase family protein
AT1G67265	DVL3/RTFL21	2.141	0.004	ROTUNDIFOLIA like 21
AT1G16410	BUS1/CYP79F1	2.086	0.000	Member of CYP79F
AT4G13770	CYP83A1/REF2	2.051	0.000	Cytochrome p450 enzyme

Table S3. List of down-regulated genes in *AtC3H3* OX under salt stress condition

Locus tag	Gene Symbol	Fold change	*p*-value	Description
AT5G20250	DIN10/RS6	0.158	0.000	Member of glycosyl hydrolase family 36
AT5G04790	AT5G04790	0.354	0.046	Transmembrane protein
AT4G37990	CAD8/ELI3-2	0.386	0.000	Aromatic alcohol:$NADP^+$
AT3G18291	AT3G18291	0.432	0.002	Hypothetical protein
AT3G44870	FAMT-L	0.440	0.015	SABATH family methyltransferase
AT4G12735	AT4G12735	0.445	0.016	Peroxisomal protein
AT3G49620	DIN11	0.458	0.001	Encodes a protein similar to 2-oxoacid-dependent dioxygenase
AT3G21720	ICL	0.472	0.008	Glyoxylate cycle enzyme isocitrate lyase (ICL)
AT5G43570	AT5G43570	0.473	0.028	Predicted to encode a PR (pathogenesis-related) peptide that belongs to the PR-6 proteinase inhibitor family
AT5G43650	bHLH92	0.488	0.046	basic helix-loop-helix (bHLH) DNA-binding superfamily protein
AT1G79485	AT1G79485	0.499	0.023	Hypothetical protein

Table S4. Biological process categories of GO of DEGs in *AtC3H3* OXs under salt stress condition

GO	Term	Number of genes	*p*-value	Expression
GO:0009625	response to insect	2	1.16E-02	Up
GO:0019758	glycosinolate biosynthetic process	2	1.51E-02	
GO:0016144	S-glycoside biosynthetic process	2	1.51E-02	
GO:0019761	glucosinolate biosynthetic process	2	1.51E-02	
GO:1901659	glycosyl compound biosynthetic process	2	3.27E-02	
GO:0019748	secondary metabolic process	3	2.99E-02	Down

Table S5. List of primers for cloning

Construct	Forward	Reverse
AtC3H3 OX	5′-ATAGTCGACATGCGA ACCCCCATGTCAGA-3′	5′-GATGGATCCTCATGG TGATGACGCATCCT-3′
sGFP-AtC3H3	5′-ATAGTCGACATGCGA ACCCCCATGTCAGA-3′	5′-GATGGATCCTCATGG TGATGACGCATCCT-3′
AtC3H3-sGFP	5′-ATAGTCGACATGCGA ACCCCCATGTCAGA-3′	5′-ATAGGATCCGTGGTG ATGACGCATCCTCAG-3′
P_{AtC3H3}::*GUS*	5′-CTGAAGCTTCTATAC CGTTGCCCATTCCG-3′	5′-GCGCTGCAGGTAAGG AAAGACGATGATCA-3′

Table S6. List of primers used for qRT-PCR and semi-qRT-PCR

Gene	Forward	Reverse	Purpose
AtC3H3	5′-GACAACCAGCTTGTGGTAAC-3′	5′-CATAGGGTGGTCGAATTTGC-3′	qRT-PCR
GAPc	5′-GTGTCCCAACCGTTGATGTC-3′	5′-TCCCTTGAGTTTGCCTTCGG-3′	qRT-PCR
RD29A	5′-CCTGAAGTGATCGATGCACC-3	5′-CAGTGGGTTTGGTGTAATCG-3	qRT-PCR
RAB18	5′-TACCAGAACCGTCCAGGAGG-3	5′-CGTACTCGTCATACTGCTGC-3	qRT-PCR
RD29B	5′-TTCTTGGCTCGGTGGTAAAC-3	5′-GGTGCCAAGTGATTGTGGAG-3	qRT-PCR
RD22	5′-GTAAACCCGGTAAAAGAACC-3	5′-TACACGAAAGGGTTTGCTCC-3	qRT-PCR
DREB2A	5′-GTGTTGCCAACGGTTCATAC-3	5′-GAGGTATTCCGTAGTTGAGG-3	qRT-PCR
DREB2B	5′-GAAGAGTCTTGTGGAACCAG-3′	5′-CCCAATACTGCTGCTCAAAC-3′	qRT-PCR
AtC3H3	5'-GACAACCAGCTTGTGGTAAC-3'	5'-CAACATCACTGGTTTCATCC-3'	semi-qRT-PCR
GAPc	5'-CACTTGAAGGGTGGTGCCAAG-3'	5'-CCTGTTGTCGCCAACGAAGTC-3'	semi-qRT-PCR
RD29A	5'-GAAACAGAGTCTGCCGTGAC-3'	5'-TGCTGCCTTCTCGGTAGAGA-3'	semi-qRT-PCR
